# A Study on Mortality Profile among Fifty Plus- (50+-) Population (FPP) of India: A 5-Year Retrospective Study at New Delhi District

**DOI:** 10.1155/2016/6403103

**Published:** 2016-03-03

**Authors:** B. L. Chaudhary, Raghvendra K. Vidua, Arvind Kumar, Amrita V. Bajaj

**Affiliations:** ^1^Department of Forensic Medicine & Toxicology, Lady Hardinge Medical College, C-604, Shaheed Bhagat Singh Marg, Connaught Place, New Delhi 110001, India; ^2^Department of Forensic Medicine & Toxicology, AIIMS, Saket Nagar, Bhopal, Madhya Pradesh 462020, India; ^3^Department of Medicine, AIIM, Saket Nagar, Bhopal, Madhya Pradesh 462020, India

## Abstract

*Objectives*. To find out the mortality profile vis-a-vis different epidemiological factors at the time of autopsy among the 50+-Population.* Material and Method*. A five-year retrospective evaluation of medicolegal records between 2006 and 2010 was done at Lady Hardinge Medical College, New Delhi.* Results*. A total of 493 (17.78%) cases belonged to 50+-Population age group out of total 2773 autopsies performed. The proportion of unidentified/unknown persons among this age group was 36.51%. The unnatural and natural causes constituted 44.62% and 55.38% cases, respectively. The unspecified pneumonitis (50.18%) was reported as the commonest cause followed by coronary artery disease and respiratory tuberculosis among natural ones and the transport accident (57.27%) followed by accidental and intentional self-poisoning and exposure to noxious substances and falls among the unnatural ones.* Conclusion*. The findings reveal that this age group most commonly dies of natural causes rather than the unnatural ones even in autopsy cases. They have definite cure with timely interventions. The study also points out the need to devise the road and home safety measures to reduce mortality among the study population.

## 1. Introduction

As per 2011 census, 19, 20, 64,349 (50.18% males and 49.82% females) people in India were of fifty plus- (50+-) age group, which made about 15.86 percent of the total population while 39,67,805 (20,96,841 males and 18,70,964 females) and 21,773 (12,391 males and 9,382 females) persons out of total fifty plus- (50+-) population used to live in National Capital Territory of Delhi and district of New Delhi, respectively [1]. India's population aged fifty plus- (50+-) is expected to double by 2050 when nearly one-third of its total population would fall in this age group, according to a US census [2]. Fifty plus- (50+-) population (FPP) taken for the present study means the persons who are at or above the age of 50 years or the persons who have already spent their 50 years of lives. This group includes ageing population comprised of those who are already elders (60 years or above) or those who are on the verge of inclusion into this group (50+). Such type of persons is usually on the declining phase for their body growth and general health.

Elderly or old age consists of ages nearing or surpassing the average life span of human beings. The boundary of old age cannot be defined exactly because it does not have the same meaning in all societies. Government of India adopted a National Policy on Older Persons in January, 1999; the policy defines “senior citizen” or “elderly” as a person who is of age 60 years or above (60+). This age group is inclusive in the fifty plus- (50+-) population age group taken for the present study.

As per survey conducted under National Sample Survey 60th round during January and June 2004, the survey estimated the number of aged persons (60+) as 829917, accounting for 5.49% of the total population of Delhi, and fifty plus- (50+-) population (FPP) was about 1877393 (12.42% of Delhi's population) [3]. The life expectancy of Indians has increased from 56.6 to 63.7 during the last two decades. With marginal success in control of communicable and infectious diseases along with improved standards of living, the number of elderly people has been on the increase. Consequently, people are living longer and elderly population has been increasing. Nearly 8% of Indian population belongs to 60+ years' age group [4].

## 2. Materials and Methods

In the present study, the medicolegal autopsy reports at Department of Forensic Medicine & Toxicology, Lady Hardinge Medical College, New Delhi (India), from 2006 to 2010, was taken into account. During this period, a total of 2773 medicolegal autopsies were performed. In the same period a total of 3,45,707 (64.43% males and 35.57% females) deaths of age group above 45 years were registered in National Capital territory of Delhi. In the same period NDMC (New Delhi Municipal Corporation) which registers all the deaths of New Delhi district has registered a total of 1,04,478 deaths (66.33% males and 33.67% females) [5]. Thus the number of cases in present study constitutes 0.80% of total registered deaths of 45+ years of age group in National Capital territory of Delhi and 2.65% of all the registered deaths of all the age groups reported by the NDMC.

Medical and other investigative data from the police requisition reports were available in each case and they provided the data about the personal details, the manner of death, and approximate time since death of the deceased while the autopsy reports provided the postmortem findings, time of death, and cause of death. The inclusion criteria were that the deceased was of 50+ years of age at the time of death. The manner of death was registered as natural, accidental, suicidal, homicidal, or unknown, after evaluation of all available information in police inquest report and postmortem examination. All deaths were further subcategorized according to the different epidemiological factors such as age, sex, year, manner and cause of death, and whether deceased was identified/known with a valid home address available or homeless and unidentified/unknown person (HUP).

## 3. Results

In the present study a total 2773 medicolegal autopsies were reported during this time period. A total of 493 (17.78%) cases were belonging to 50+ population age group. Out of total cases of 50+ population age group, the males were 438 cases (88.84%) and females were 55 cases (11.16%). The male to female cases ratio was 7.96 : 1. The deaths due to unnatural and natural events constituted 220 (44.62%) and 273 (55.38%) cases, respectively (Table 1). Out of 493 cases, 180 (36.51%) cases were unidentified/unknown persons (HUPs) or persons without any valid home address whose identity could not be established at the time of autopsy. The male and female deaths in unidentified/unknown persons constituted 158 cases (87.77%) and 22 cases (12.22%), respectively.

**Figure 1:**
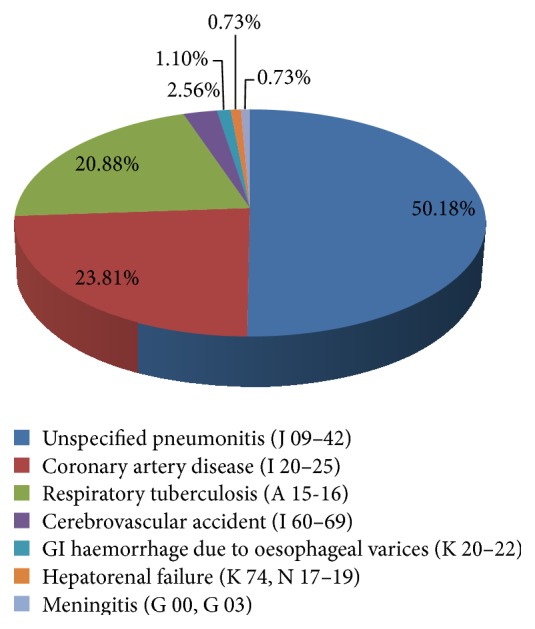
Distribution of natural causes of death among 50+ population.

There were 273 (55.38%) deaths due to natural causes and unspecified pneumonitis contributed to the maximum number of 137 (50.18%) of cases followed by coronary artery disease 65 cases (23.81%) and respiratory tuberculosis 57 cases (20.88%) among them (Figure 1). Out of 273 cases, 155 (56.78%) cases were homeless unknown/unidentified persons (HUPs) and remaining 119 (43.22%) cases were identified or having a valid home addresses. Coronary artery disease was major cause of death in 60 cases (38.71%) among identified/known or persons having a valid home address but the unspecified pneumonitis was major cause of death in 105 (67.74%) cases of homeless unidentified/unknown persons (HUPs).

Respiratory tuberculosis was reported as cause of death in 40 cases (25.81%) of homeless unknown/unidentified and in 17 cases (14.29%) of identified/known persons having a valid home address. The male and female ratio among the persons who died of natural deaths was 12.65 : 1. Unspecified pneumonitis (*n* = 137, 50.18%) was the major health problem in 50+ population and was a leading cause of natural deaths. The male-to-female ratio for unspecified pneumonitis was 12.7 : 1. After unspecified pneumonitis, respiratory tuberculosis was the second leading cause of death among natural deaths in 57 (20.88%) cases.

**Figure 2:**
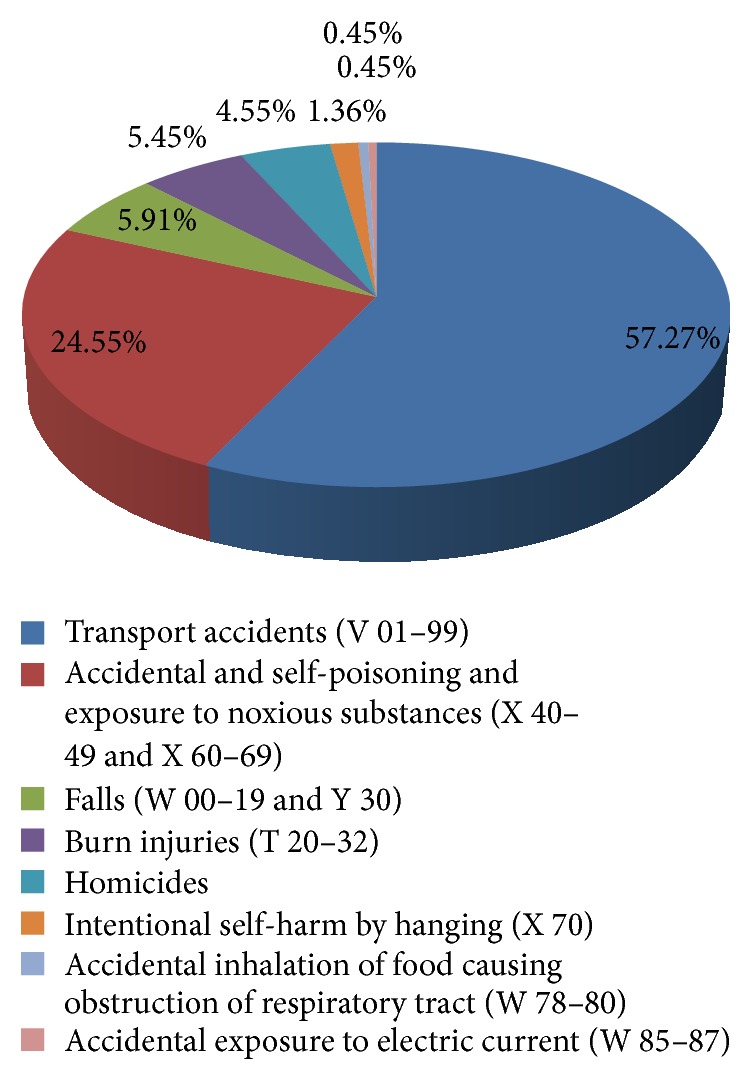
Distribution of unnatural causes of death among 50+ population.

In the 50+ population, the accidental and intentional self-poisoning and exposure to noxious substances were reported as the second leading cause of unnatural deaths and responsible for deaths in 54 (34.55%) cases. The organophosphates which is very commonly used agricultural pesticide in India was found as the commonest poison responsible for deaths due to exposure of poisons and noxious substances based upon police inquest report, postmortem examination awaiting viscera lab analysis report. The financial burden and family dispute were the most common reason for committing suicide as per the police inquest report. All the cases had occurred at home when the person was alone in absence of immediate family members. The homicides constituted a total of 10 deaths among the unnatural deaths and the assault by firearm weapon (40% cases) was the commonest means used (Figure 2).

**Figure 3:**
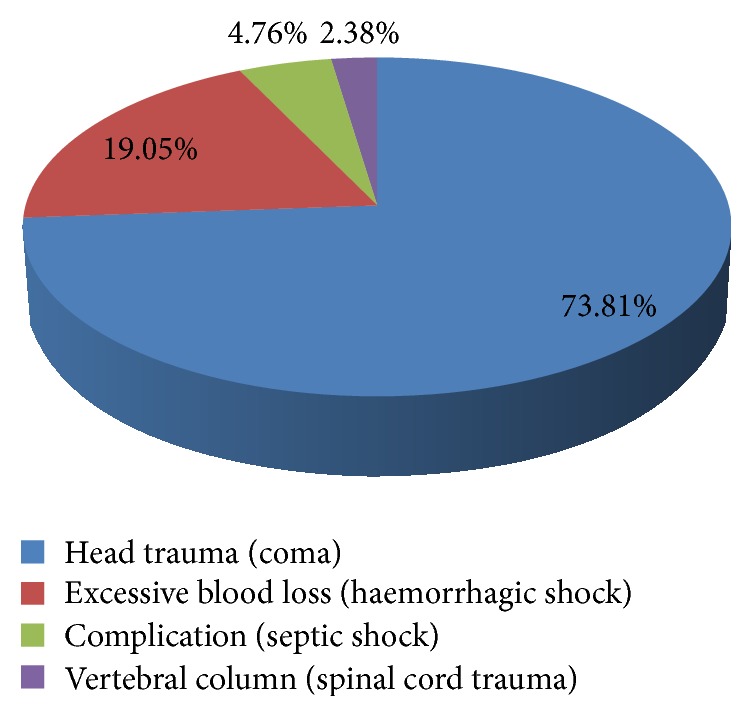
Mode of death in transport accidents among 50+ population.

Among 220 unnatural deaths the leading contributor to death was the transport accidents (*n* = 126, 57.27%) in 50+ population and out of that, in 16 (12.70%) cases, the identity of the person could not be established. All of the road traffic accidents had occurred within the New Delhi district of the city and majority (*n* = 41 cases, 32.54%) during the evening hours between 6 pm and 12 midnight followed by morning hours (*n* = 33 cases, 26.19%) between 6 am and 12 at noon. Pedestrians was the most commonly affected group and nearly half of deaths were due to be hit by heavy vehicles like buses, cars, trucks, and van/jeeps. The head injury (coma) was found as the commonest mode of death in 93 (73.81%) cases (Figure 3).

## 4. Discussion

In the present study, 493 (17.78%) cases were of 50+ population age group and the male-to-female ratio among them was 7.96 : 1. As per the National Sample Survey organization, 39% of the elderly of 60+ age are likely to be suffering from one or the other health problem [3]. It is estimated that 1.7% and 1% suffer from visual disability, 1.5% and 1.3% hearing, and 2.7% and 2.8% locomotor difficulties in rural and urban areas, respectively [4]. Data from National Crime Records Bureau–2006 [6] indicate that 34,594, 60+ individuals lost their life due to an injury. Some epidemiological surveys on aged population indicate that 1.7% was affected with injuries [7]. Previous studies from NIMHANS on traumatic brain injuries and road traffic injuries reveal that 5–8% of deaths and hospitalisations are among 60+ people [8]. Among the various types of injuries, road traffic injuries and falls are found to be the leading causes of injury. One-year (2007) data from Bengaluru injury surveillance programme showed that 360 individuals of 60+ years age group died and 2643 of 60+ years age group are individuals suffering from various forms of injuries brought to hospitals. Majority of those killed or hospitalized belonged to middle-income families [9]. The male-to-female distribution was almost equal with 198 men and 162 women in contrast to the present study with 438 male cases to 55 female cases.

In the present study, 50+ population cases accounted for 17.81% (55.46% natural deaths and 44.53% unnatural deaths) of the total medicolegal autopsies conducted in the mortuary of Lady Hardinge Medical College (LHMC), New Delhi. In the study by Ince et al. [10], this rate was 7.8%. Homicide and suicide origins accounted for 18.9% which can be considered as higher compared with the literature. In Osaka [11], during 1994–1998, this rate was 13.2%. In another study by Collins and Presnell [12], this rate was 12.4%. In the present study, stabbing was the most common method of homicide, while gunshot was the most common method in other studies [13, 14]. In the present study, male/female proportion was 3 : 1 and this was consistent with other Turkish studies [10, 15]. Psychiatric illnesses were associated with suicide tendency [11]. In the elderly, 19% of the female and 9% of the male victims had a history of previous suicide attempts [16]. In the present study, poisoning (47.1%) was the most common method of suicide unlike hanging in the study held in Aydin [17, 18]. Hanging is the most frequent suicide method used by the elderly also in Austria [19] and many other countries [10, 18]. Use of poison was very common in the present study as also in Pritchard and Hansen's study [18]. The most common wound site was the head region in road traffic accidents consistently with other studies [18, 20]. The 60+ victims had a higher rate of chest injuries and the commonest method of suicide was hanging followed by organophosphate poisoning [21].

## 5. Limitations of the Study

Consider

1. There were a large number of deceased persons in this study whose identity could not be established till the time of autopsy which adversely affected the amount of reliability and details of history obtained.
2. Authors could not find any other such type of studies conducted in this particular age group, so not much data was available for comparison. Therefore, in the present study a comparison was made with the mortality profile of age group of sixty plus- (60+-) population (SPP) as many of the studies have been conducted in this group in the past and it is also inclusive in fifty plus- (50+-) population (FPP) age group.
3. The Lady Hardinge Medical College is located in the National Capital Territory region of the country. The demographic profile of population is much different in such type of metropolitan city compared to the other cities or villages of India so the results of the studies cannot be generalised for the similar age group population of rest of the country but of course it may be taken as a comparable reference point for similar kind of metropolitan cities.
4. The detailed lab analysis report regarding poisoning cases was not available on time and in most of the cases of unspecified pneumonitis and respiratory tuberculosis the histopathological investigations were not done and the diagnosis was made on the basis of gross examination of lung and its cut sections.

## 6. Conclusion

With the growth of ageing population in almost each and every society of the world and consequent rise in their mortality, it is important to study their mortality profile to find out various reasons responsible for their untimely natural and unnatural mortality because of emergence of certain age related risk factors for devising appropriate intervention strategies. In this study the major factors responsible for mortality among them were found out as unspecified pneumonitis, coronary artery disease, and respiratory tuberculosis among natural causes and transport accidents, accidental and intentional self-exposure to poisoning, and falls among unnatural causes. The interventions need to be integrated in a comprehensive manner with the focus on provision of early diagnosis and treatment along with the modification of risk factors for natural causes and effective preventive strategies along with the timely treatment and enforcement of adequate road safety measures like use of helmet to reduce the occurrence of head injuries in transport accidents for unnatural causes. The findings clearly reveal that this age group most commonly dies of natural causes like pulmonary and cardiovascular diseases rather than the unnatural ones even in autopsy cases. They have definite cure with timely interventions and that will also be helpful to reduce the spread of communicable infections to the rest of the population. The home safety measures should also be devised to reduce their accidental and intentional self-exposure to poisoning and fall injuries to reduce the mortality in fifty plus- (50+-) population (FPP) at New Delhi district in India.

## Figures and Tables

**Table 1:** Year- and sex-wise distribution of natural and unnatural deaths in 50+ population.

Year	Natural deaths		Unnatural deaths		Total
	Male	Female	Male	Female	
2006	49	4	25	8	86
2007	27	0	47	11	85
2008	52	3	50	4	109
2009	41	5	27	6	79
2010	84	8	36	6	134
Total	**253**	**20**	**185**	**35**	**493**
